# Modelling the initial epidemic trends of COVID-19 in Italy, Spain, Germany, and France

**DOI:** 10.1371/journal.pone.0241743

**Published:** 2020-11-09

**Authors:** Kai Wang, Lin Ding, Yu Yan, Chengguqiu Dai, Minghan Qu, Dong Jiayi, Xingjie Hao

**Affiliations:** Department of Epidemiology and Biostatistics, Key Laboratory for Environment and Health, School of Public Health, Tongji Medical College, Huazhong University of Science and Technology, Wuhan 430030, China; Xavier University, UNITED STATES

## Abstract

The Coronavirus Disease 2019 (COVID-19) has fast spread to over 200 countries and regions worldwide since its outbreak, while in March, Europe became the emerging epicentre. In this study, we aimed to model the epidemic trends and estimate the essential epidemic features of COVID-19 in Italy, Spain, Germany, and France at the initial stage. The numbers of daily confirmed cases and total confirmed cases were extracted from the Coronavirus disease (COVID-19) situation reports of WHO. We applied an extended Susceptible-Exposed-Infectious-Removed (SEIR) model to fit the epidemic trend and estimated corresponding epidemic features. The transmission rate estimates were 1.67 (95% credible interval (CrI), 1.64–1.71), 2.83 (2.72–2.85), 1.91 (1.84–1.98), and 1.89 (1.82–1.96) for Italy, Spain, Germany, and France, corresponding to the basic reproduction numbers (*R*_0_) 3.44 (3.35–3.54), 6.25 (5.97–6.55), 4.03 (3.84–4.23), and 4.00 (3.82–4.19), respectively. We found Spain had the lowest ascertainment rate of 0.22 (0.19–0.25), followed by France, Germany, and Italy of 0.45 (0.40–0.50), 0.46 (0.40–0.52), and 0.59 (0.55–0.64). The peaks of daily new confirmed cases would reach on April 16, April 5, April 21, and April 19 for Italy, Spain, Germany, and France if no action was taken by the authorities. Given the high transmissibility and high covertness of COVID-19, strict countermeasures, such as national lockdown and social distancing, were essential to be implemented to reduce the spread of the disease.

## Introduction

Since the first outbreak at the end of December 2019 in Wuhan, China, the Coronavirus Disease 2019 (COVID-19) has fast spread to over 200 countries and regions worldwide, causing more than 376,320 deaths of 6,194,533 confirmed cases by June 2 [1]. To mitigate the pandemic [2], many countries have declared border closures, with a partial suspension of navigation. Although a serial of mandated measures implemented in China (*e*.*g*., city cordon sanitaire in Hubei) have delayed the epidemic situation to spread to global scope by 3 to 5 days [3], it still failed to raise other countries’ awareness of the high transmissibility and serious perniciousness of the epidemic at the early stage.

China has successfully suppressed the epidemic after imposing strict control measures for approximately two months since the epidemic outbreak, while Europe and America emerged as the new vortex centre. In the initial stage of the epidemic during late January in Europe, the top transmission hotspots were Italy, Spain, Germany, and France, and they are still at the forefront [1]. Case increments in these countries presented exponential-like trends during the early stage with no powerful countermeasures taken. And realistic data has proven that the pandemic is certainly posing a major threat to human health as well as social operation. Modelling the dynamic epidemic transmission using data from the early stage is of indubitable importance for characterizing the transmission parameters, tracing the trajectory of the epidemic, and making evaluation for control measures.

Given the severity of COVID-19, clarifying the situation and predicting the trend of the epidemic are of absolute significance for the transmission reduction, the coordination, and allocation of medical resources, and eventually, for lives saving. There have been a number of studies using Susceptible-Exposed-Infectious-Removed (SEIR) models aiming at countries with severe epidemics since the outbreak [4–6], while studies of the epidemic model and forecaster for European countries were relatively less. A model applied to hospital and death data in France indicated that the lockdown reduced the reproduction number from 2.90 to 0.67, suggesting a 77% reduction in transmission [7]. Linka, K., et al. [8] modelled the epidemiology of the COVID-19 outbreak in all 27 states of the European Union using a SEIR model with conventional settings. However, the distinctive role of asymptomatic and mild-symptomatic cases, which could be pivotal sources of infection [9,10], has not been deliberated.

Noticing the limitations of the aforementioned studies, we applied an extended SEIR model, accounting for population movement, unascertained cases, and hospitalization [11], to model the epidemic at the initial stage of the four most severely affected European countries (*i*.*e*., Italy, Spain, Germany, and France). The parameters, including transmission rate, ascertainment rate, and basic reproduction number, were estimated by fitting the data of daily confirmed cases using the Markov Chain Monte Carlo (MCMC) Method. We then predicted the epidemic trends under different intensities of countermeasures in each country.

## Materials and methods

### Data source

Cases of COVID-19 in Italy, Spain, Germany, and France at the initial stage were extracted from the daily situation reports of the World Health Organization (WHO) [12], including the numbers of confirmed cases and total confirmed cases per day. To better reflect the epidemic trends of COVID-19 in these countries, the start and end date of analysis were selected according to the outbreak situation of each country. For Italy, we chose to analyse data from February 22, when the first domestic case was confirmed [13], to March 10, 2020, when the Italian government announced nationwide city closures [14]. For Spain, we chose to start from February 27, when the first case of local exposure was confirmed [15], to March 14, 2020, when the Spanish government implemented blockade nationwide [16]. Data for France was extracted from February 27 to March 14 because the number of reported new cases once stopped growing before the start date, and French authority declared restriction on mass gathering countrywide on March 14 [17]. Finally, the German data was extracted from February 26, when two cases associated with the Italian Carnival were confirmed, to March 14, 2020, for the consistency with other countries. For Germany, we did not choose the date when the first case appeared as the start point because all the reported cases before February 26 were pointed to a local company, and with strict quarantine [18].

### Statistical analysis

**Fig 1:**
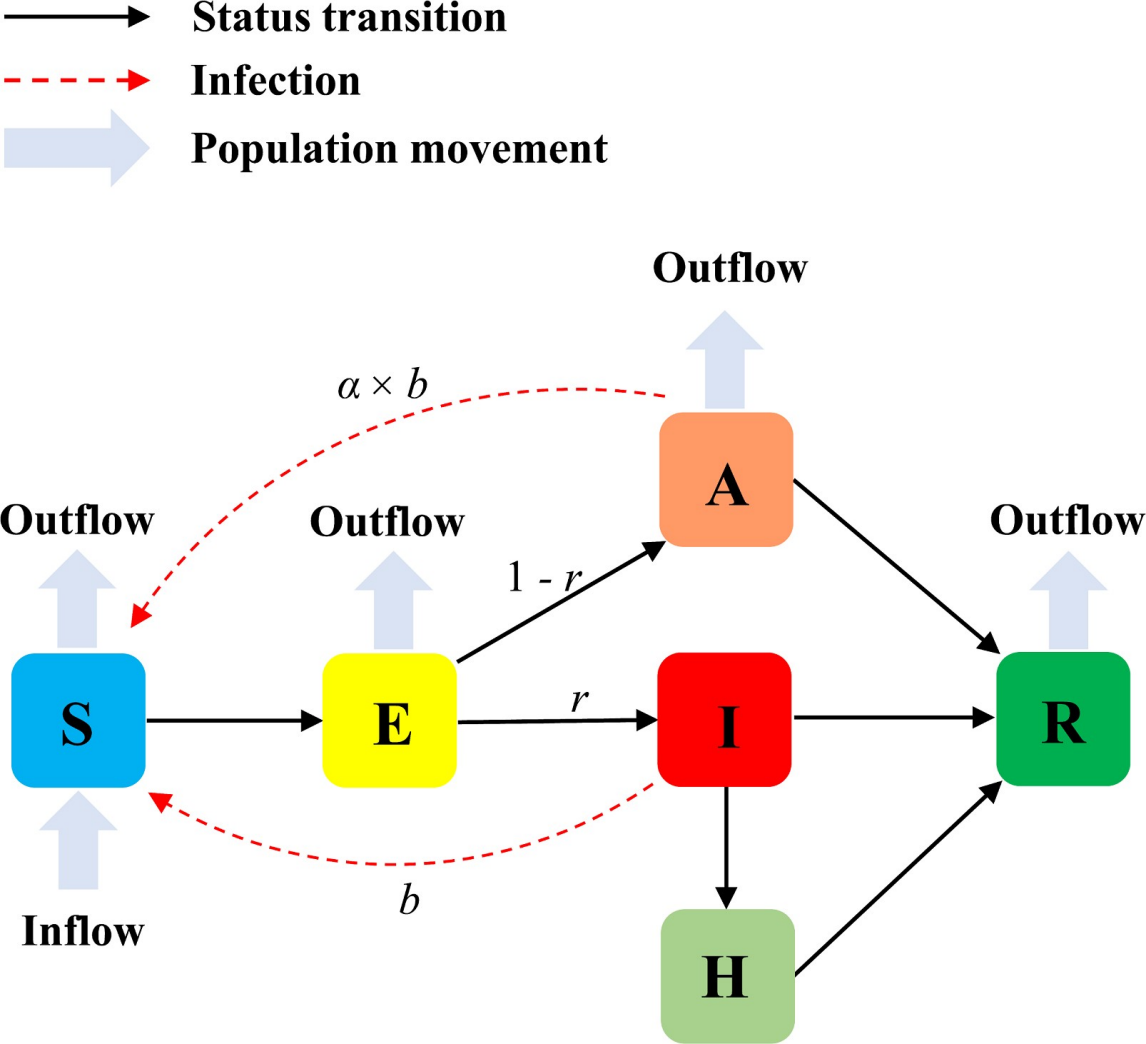
Illustration of the extended SEIR model. The population was divided into six compartments: S (susceptible), E (latent), I (reported infectious), A (unascertained infectious), H (hospitalized), and R (removed including both recovered and died cases). *r* was the ascertainment rate; *b* was the transmission rate of ascertained cases; *α* was the ratio of transmission rate between unascertained and ascertained cases.

We applied an extended SEIR model, taking account of population movement, unascertained cases, and hospitalization, to fit the epidemic trends and estimate the essential epidemic features of COVID-19 at the initial stage. The model assumed a constant population size, ignored all demographic changes in the population (*i*.*e*., births, or deaths other than from COVID-19), and has been detailed in our previous study [11]. In brief, this model divided the population into six compartments including susceptible individuals (S), latent cases (E), ascertained cases (I), unascertained cases (A), hospitalized cases (H), and removed individuals (R), which included both recovered and dead cases (**Fig 1**). Here, unascertained cases included asymptomatic cases and those with mild symptoms who could recover without seeking medical care and thus were not reported to authorities. This model assumed that only those seeking medical care would be reported and quarantined by hospitalization. Dynamics of these six compartments across time were described by the following set of ordinary differential equations:

$$\frac{dS}{dt}=-\frac{bS(I+\alpha A)}{N}+n-\frac{nS}{N-I-H}$$

$$\frac{dE}{dt}=\frac{bS(I+\alpha A)}{N}-\frac{E}{D_{e}}-\frac{nE}{N-I-H}$$

$$\frac{dI}{dt}=\frac{rE}{D_{e}}-\frac{I}{D_{q}}-\frac{I}{D_{i}}$$

$$\frac{dA}{dt}=\frac{(1-r)E}{D_{e}}-\frac{A}{D_{i}}-\frac{nA}{N-I-H}$$

$$\frac{dH}{dt}=\frac{I}{D_{q}}-\frac{H}{D_{h}}$$

$$\frac{dR}{dt}=\frac{I+A}{D_{i}}+\frac{H}{D_{h}}-\frac{nR}{N-I-H}$$

Where *b* was the transmission rate, defined as the number of individuals that an ascertained case can infect per day; *α* was the ratio of the transmission rate of unascertained over ascertained cases; *r* was ascertainment rate; *D*_*e*_ and *D*_*i*_ were the latent and infectious periods; *D*_*q*_ was the duration from illness onset to hospitalization; and *D*_*h*_ was the hospitalization period. The daily cases data was assumed to follow the Poisson distribution under the SEIR model.

Initial states of the model and parameter settings were described in **Tables 1A, 1B and 2**. We assumed a constant population size (*N*) with equal daily inbound and outbound travellers (*n*) for each country in the SEIR model as previous studies [19]. The average numbers (*n*) of daily inbound and outbound travellers between January 20 and March 2, 2020, were extracted from the Official Airlines Guide (OAG) database [20]. We assumed the same transmissibility between unascertained and ascertained cases (*α* = 1). The mean latent period (*D*_*e*_)and infectious period (*D*_*i*_) were set to 5.2 and 2.3 days [21], respectively, assuming the latent period equal to the incubation period, and the infectious period equal to the difference between the serial interval and the incubation period [19]. We chose the duration from onset to hospitalization (*D*_*q*_) as 10 days, considering the reported mean *D*_*q*_ of 9.1 days for initial cases in Wuhan [21]. The hospitalization period (*D*_*h*_) was set to 30 days for the four countries, but this parameter had no effect on our fitting procedure and the final parameter estimates.

**Table 1:** A. Initial state of the extent SEIR model for the main analysis in Italy and Spain. **B.** Initial state of the extent SEIR model for the main analysis in Germany and France.

Variable	Meaning	Italy		Spain	
		Value	Note	Value	Note
*S*	Number of susceptible individuals	61,641,231	*S* = *N*—*E*—*I* -*R* -*H*—*A*	46,748,976	*S* = *N*—*E*—*I* -*R* -*H*—*A*
*E*	Number of latent cases	638	Twice of the number of cases with confirmed from Feb. 22 to 26, 2020	204	Twice of the number of cases with confirmed from Feb. 28 to Mar. 3, 2020
*I*	Number of ascertained infectious cases	1	Number of cases with confirmed before Feb. 22, 2020 minus *H*(0)	10	Number of cases with confirmed before Feb. 28, 2020 minus *H*(0)
*A*	Number of unascertained infectious cases	1	Assume *A*(0) = *I*(0)	10	Assume *A*(0) = *I*(0)
*H*	Number of hospitalized cases	3	Number of cases reported by Feb. 21, 2020	2	Number of cases reported before Feb. 27, 2020
*R*	Number of removed individuals	0	Number of recovered patients by Feb. 21, 2020	0	Number of recovered patients by Feb. 27, 2020
**Variable**	**Meaning**	**Germany**		**France**	
		**Value**	**Note**	**Value**	**Note**
*S*	Number of susceptible individuals	82,927,680	*S* = *N*—*E*—*I* -*R* -*H*—*A*	65,228,125	*S* = *N*—*E*—*I* -*R* -*H*—*A*
*E*	Number of latent cases	222	Twice of the number of cases with Onset from Feb. 27 to Mar. 2,2020	346	Twice of the number of cases with Onset from Feb. 28 to Mar. 3,2020
*I*	Number of ascertained infectious cases	2	Number of cases with onset before Feb. 27,2020 minus *H*(0)	6	Number of cases with onset before Feb. 28,2020 minus *H*(0)
*A*	Number of unascertained infectious cases	2	Assume *A*(0) = *I*(0)	6	Assume *A*(0) = *I*(0)
*H*	Number of hospitalized cases	2	Number of cases reported by Feb. 26,2020	12	Number of cases reported before Feb. 27,2020
*R*	Number of removed individuals	14	Number of recovered patients by Feb. 26,2020	0	Number of recovered patients by Feb. 27,2020

**Table 2:** Parameter values of the extent SEIR model for the main analysis.

Parameter	Meaning	Italy	Spain	Germany	France
*b*	Transmission rate of ascertained cases	*b*	*b*	*b*	*b*
*r*	Ascertainment rate	*r*	*r*	*r*	*r*
*α*	Ratio of transmission rate between unascertained and ascertained cases	1	1	1	1
*D*_*e*_	Latent period	5.2	5.2	5.2	5.2
*D*_*i*_	Infectious period	2.3	2.3	2.3	2.3
*D*_*q*_	Duration from illness onset to hospitalization	10	10	10	10
*D*_*h*_	Hospitalized period	30	30	30	30
*N*	Population size	60,431,283	46,749,202	82,927,922	65,228,495
*n*	Daily inbound and outbound size	250,000	360,000	380,000	260,000

We estimated the transmission rate (*b*), ascertainment rate (*r*), and their 95% Credible Intervals (CrIs) by MCMC with Metropolis-Hastings algorithm and non-informative flat priors. We set a burn-in period of 200,000 iterations and continued to run 1,000,000 iterations with a sampling step size of 100 iterations. The basic reproduction number *R*_*0*_, defined as the expected number of secondary cases infected by a primary case at time *t*, was calculated as

$$R_{0}=\frac{D_{i}b}{A+I}(\alpha A+\frac{D_{q}I}{D_{i}+D_{q}})$$

And we took the mean within the initial period as the estimate of *R*_*0*_ for each country. Based on the fitted model, we then preliminarily predicted the epidemic trend from the date when countermeasures were taken to March 31 and predicted the time when the peaks of new cases would arrive under incremental intensities of countermeasures (1) no countermeasures that the transmission rates were kept as *b*, (2) ordinary countermeasures such as handwashing, mask-wearing [22] that decreased the transmission rates to 0.75*b*, (3) strong countermeasures such as social distancing [23] that decreased the transmission rates to 0.50*b* and (4) very strong intensities such as blockade [24] that decreased the transmission rates to 0.25b.

Since multifarious assumptions were made on the initial parameter settings, we conducted a series of sensitivity analyses to determine whether the results would be affected substantially when these assumptions deviated from the truth. Unless mentioned, each sensitivity analysis’s parameter values and initial states were the same as the main analysis.

(S1) Decrease the latent period (*D*_*e*_) to 4.6 days [25], and therefore *E*(0) = 452, 204, 207, and 346 for Italy, Spain, Germany, and France, respectively;

(S2) Increase the latent period (*D*_*e*_) to 6 days [26], and therefore *E*(0) = 794, 278, 278, and 388 for Italy, Spain, Germany, and France, respectively;

(S3) Double the infectious period (*D*_*i*_) to 4.6 days;

(S4) Assume that the transmission rate of unascertained cases is half of the ascertained cases by setting *α* = 0.5;

(S5) Increase the ratio of unascertained to ascertained cases in the initial state *γ* = *A*(0)/*I*(0) to 2 by setting *A*(0) = 2, 20, 4, 12 and *E*(0) = 957, 306, 333, 519 for Italy, Spain, Germany, and France, respectively.

(S6) Decrease the hospitalization period (*D*_*h*_) to 15 days;

(S7) Decrease the duration from onset to hospitalization (*D*_*q*_) to 5 days;

(S8) Increase the duration from onset to hospitalization (*D*_*q*_) to 15 days.

### Ethics approval

The ethics approval was considered exempt because all data was collected from publicly available resources and did not contain any personal information.

## Results

**Fig 2:**
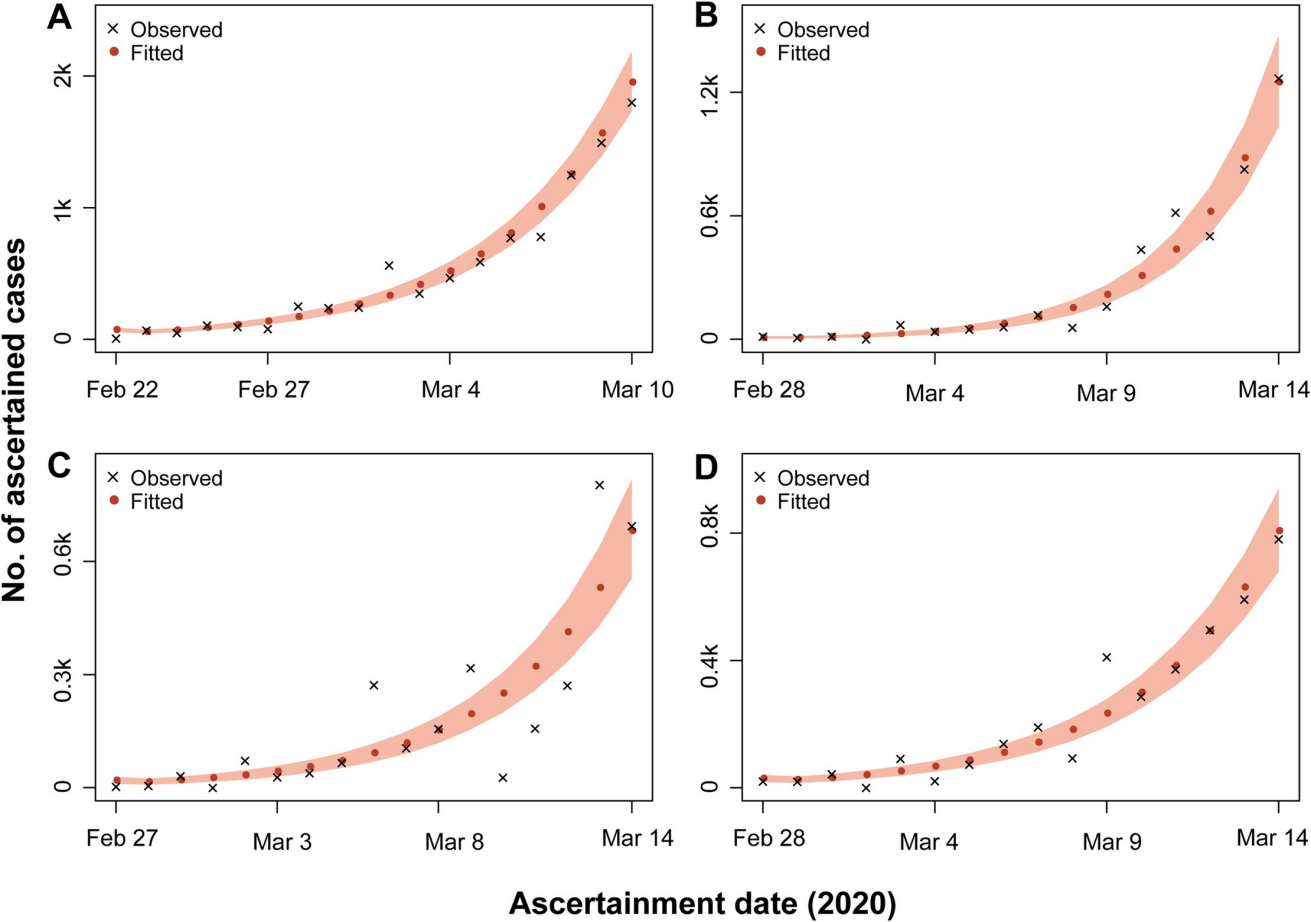
Observed and fitted number of ascertained individuals. (A) Italy; (B) Spain; (C) Germany; (D) France. The shaded areas are 95% CrIs of the predicted values.

These data represented the initial stage of COVID-19 spreading in these countries, as the trends clearly showed that the numbers of confirmed cases are still climbing (**Fig 2**). The extended-SEIR model fitted the observed data of different countries well, except for that of Germany, whose results showed a relatively obvious fluctuation away from the supposed exponential distribution trend (**Fig 2**). Apart from an obviously high transmission rate of 2.83 (95% CrI, 2.72–2.95) observed in Spain, the transmission rates of the remaining three countries were relatively consistent as 1.67 (1.64–1.71), 1.91 (1.84–1.98), and 1.89 (1.82–1.96) for Italy, Germany, and France, respectively (**Table 3**). Italy had the largest ascertained rate of 0.59 (0.55–0.64), while Spain had the lowest ascertained rate of 0.22 (0.19–0.25), and Germany and France in the middle of 0.46 (0.40–0.52) and 0.45 (0.40–0.50), respectively (**Table 3**). Given the estimated transmission rate and ascertained rate, the *R*_0_s of the initial stage for Italy, Spain, Germany, and France were 3.44 (3.35–3.54), 6.26 (5.98–6.56), 4.03 (3.84–4.23), and 4.00 (3.82–4.19), respectively (**Table 3**).

**Table 3:** Estimated *b*, *r*, and *R*_*0*_ for main and sensitivity analyses.

Country	Parameter	Analysis								
		Main	S1	S2	S3	S4	S5	S6	S7	S8
Italy	*b*	1.67 (1.64–1.71)	1.60 (1.57–1.62)	1.80 (1.77–1.84)	1.20 (1.18–1.23)	2.15 (2.05–2.25)	1.63 (1.60–1.67)	1.67 (1.64–1.71)	1.78 (1.76–1.81)	1.63 (1.60–1.67)
	*r*	0.59 (0.55–0.64)	0.75 (0.70–0.80)	0.54 (0.50–0.58)	0.66 (0.62–0.71)	0.59 (0.55–0.63)	0.40 (0.37–0.43)	0.59 (0.55–0.64)	0.58 (0.55–0.63)	0.60 (0.56–0.64)
	*R*_*0*_	3.44 (3.35–3.54)	3.17 (3.09–3.26)	3.75 (3.65–3.86)	4.44 (4.28–4.61)	3.34 (3.25–3.43)	3.50 (3.40–3.59)	3.44 (3.35–3.54)	3.42 (3.32–3.52)	3.47 (3.38–3.56)
Spain	*b*	2.83 (2.72–2.95)	2.60 (2.50–2.71)	3.12 (3.00–3.26)	2.15 (2.05–2.25)	4.74 (4.44–5.05)	2.81 (2.69–2.93)	2.83 (2.72–2.95)	2.89 (2.78–3.00)	2.81 (2.69–2.93)
	*r*	0.22 (0.19–0.25)	0.20 (0.17–0.23)	0.19 (0.17–0.22)	0.25 (0.21–0.28)	0.21 (0.18–0.24)	0.15 (0.13–0.17)	0.22 (0.19–0.25)	0.22 (0.19–0.25)	0.22 (0.19–0.26)
	*R*_*0*_	6.26 (5.98–6.56)	5.77 (5.51–6.03)	6.94 (6.62–7.26)	9.14 (8.65–9.67)	6.13 (5.83–6.44)	6.30 (6.01–6.59)	6.26 (5.98–6.55)	6.24 (5.94–6.23)	6.28 (5.98–6.57)
Germany	*b*	1.91 (1.84–1.98)	1.77 (1.71–1.84)	2.10 (2.02–2.18)	1.39 (1.33–1.45)	2.68 (2.48–2.88)	1.87 (1.80–1.95)	1.91 (1.84–1.98)	2.00 (1.94–2.06)	1.88 (1.80–1.95)
	*r*	0.46 (0.40–0.52)	0.42 (0.36–0.47)	0.52 (0.45–0.59)	0.52 (0.45–0.59)	0.46 (0.40–0.52)	0.31 (0.27–0.36)	0.46 (0.40–0.52)	0.46 (0.40–0.52)	0.46 (0.40–0.52)
	*R*_*0*_	4.03 (3.84–4.23)	3.77 (3.60–3.95)	4.38 (4.17–4.61)	5.42 (5.08–5.78)	3.90 (3.72–4.09)	4.08 (3.89–4.28)	4.03 (3.84–4.23)	4.01 (3.81–4.21)	4.06 (3.87–4.26)
France	*b*	1.89 (1.82–1.96)	1.75 (1.69–1.81)	2.06 (1.99–2.14)	1.37 (1.32–1.43)	2.68 (2.49–2.87)	1.85 (1.78–1.93)	1.89 (1.82–1.96)	1.97 (1.91–2.03)	1.85 (1.78–1.93)
	*r*	0.45 (0.40–0.50)	0.40 (0.36–0.45)	0.45 (0.40–0.50)	0.50 (0.44–0.55)	0.44 (0.39–0.49)	0.30 (0.27–0.33)	0.45 (0.40–0.50)	0.44 (0.39–0.49)	0.45 (0.40–0.50)
	*R*_*0*_	4.00 (3.82–4.19)	3.74 (3.58–3.91)	4.37 (4.17–4.58)	5.40 (5.10–5.71)	3.87 (3.70–4.06)	4.04 (3.87–4.22)	4.00 (3.82–4.18)	3.98 (3.80–4.17)	4.02 (3.84–4.21)

**Notes**: the estimates were displayed as mean (95% Credible Intervals) based on 1,000,000 Markov Chain Monte Carlo iterations.

**Fig 3:**
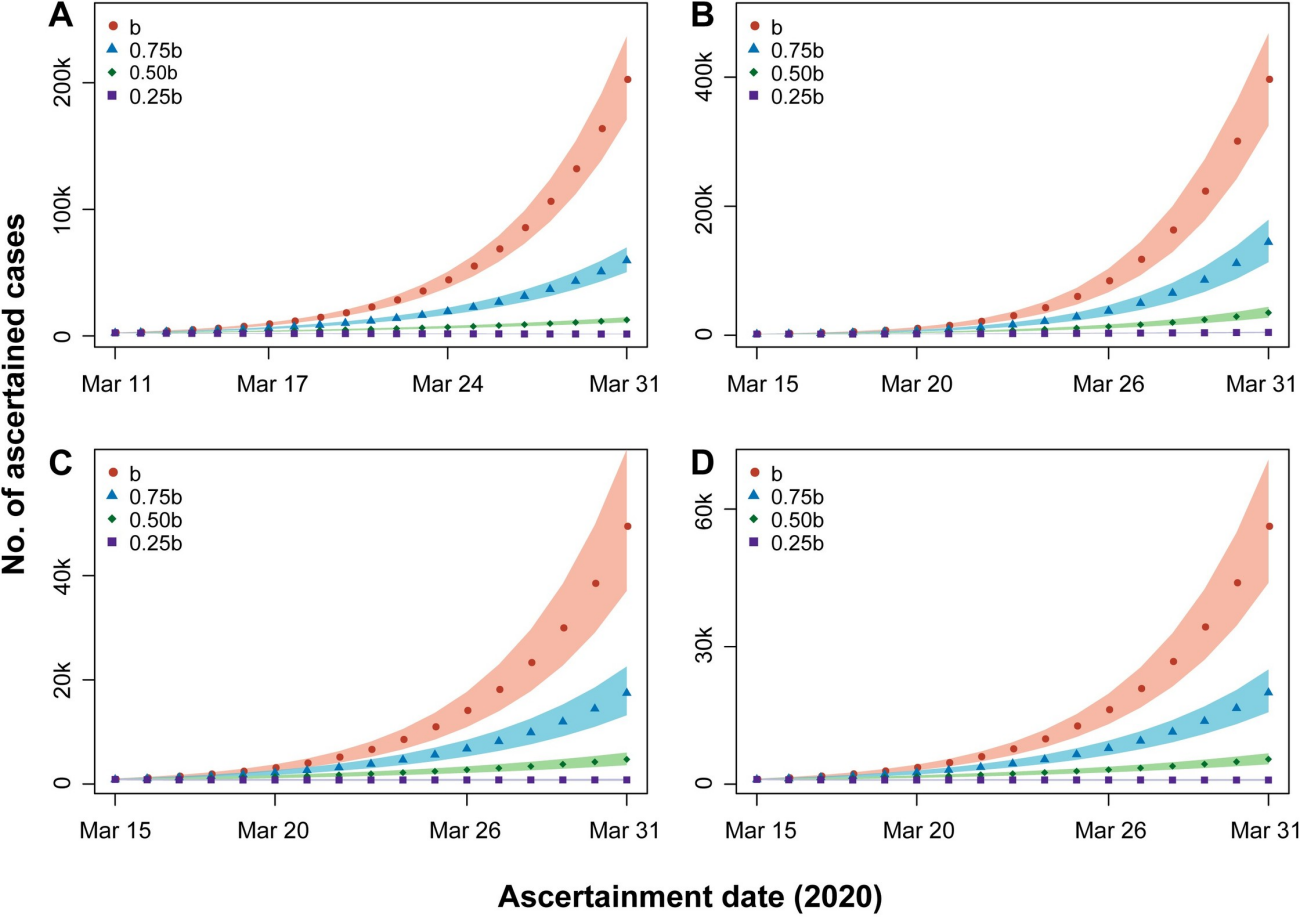
Predicted number of ascertained individuals by March 31 under different transmission rate (*b*, 0.75*b*, 0.50*b*, 0.25*b*) assumptions. (A) Italy; (B) Spain; (C) Germany; (D) France. The shaded areas are 95% CrIs of the predicted values.

**Fig 4:**
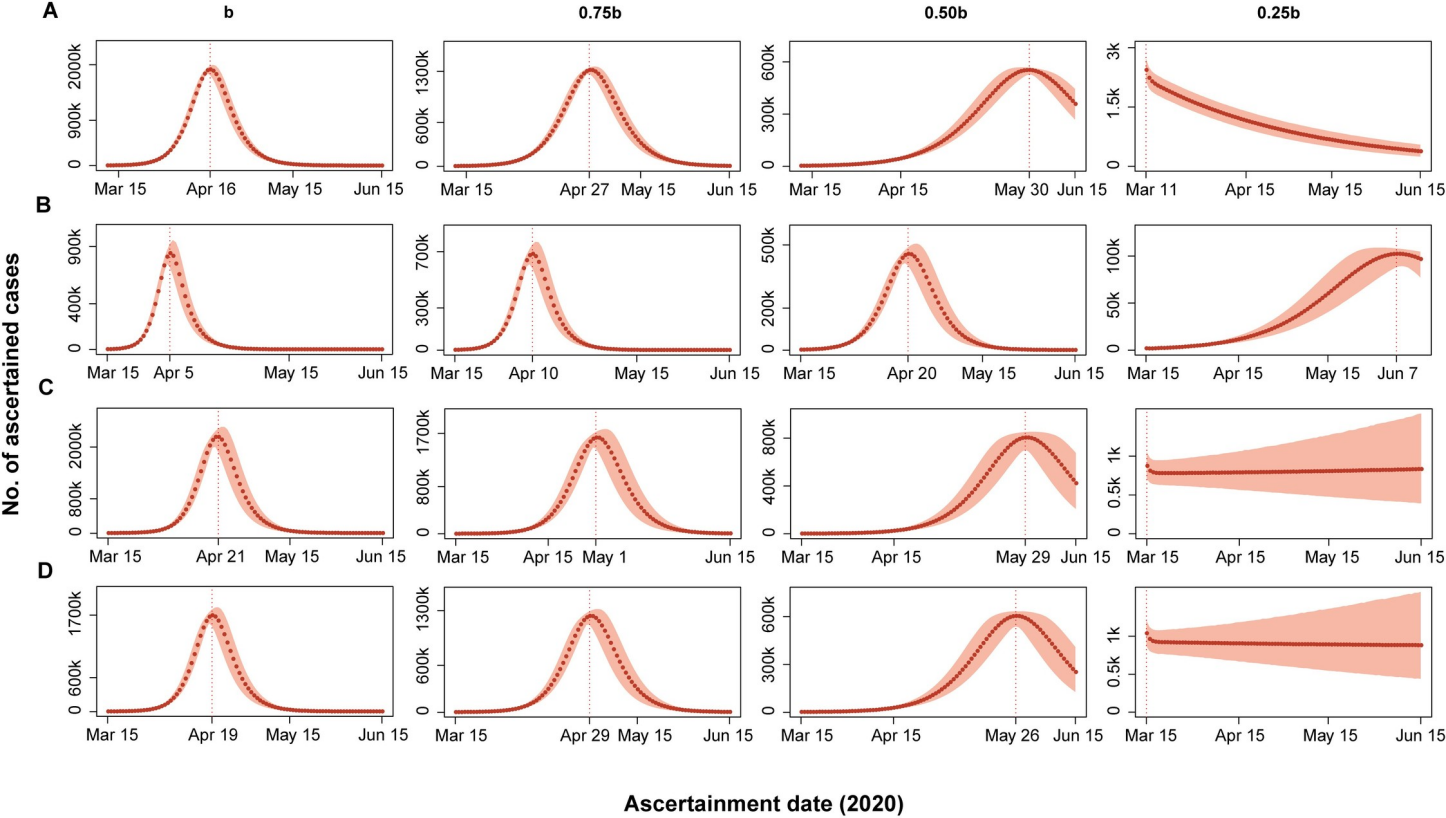
Predicted the peaks of ascertained cases under the assumptions of different transmission rates (*b*, 0.75*b*, 0.50*b*, 0.25*b*). (A) Italy; (B) Spain; (C) Germany; (D) France. The shaded areas are 95% CrIs of the predicted values.

We assumed 0%, 25%, 50%, 75% reduction of the transmission rate, reflecting different intensities and types of interventions, to predict the number of ascertained cases from the date when strict countermeasures were taken to March 31. If no action (0% reduction of the transmission rate) was taken, the ascertained number would rise to 202,258 (171,297–236,761), 395,296 (323,235–466,790), 49,353 (37,043–64,131), and 56,174 (43,784–70,565) for Italy, Spain, Germany, and France by March 31, respectively (**Fig 3**). However, if authorities took lockdown (75% reduction), the number would be 1,520 (1,296–1,759), 4,362 (3,446–5,418), 781 (605–986), and 909 (732–1,108), respectively (**Fig 3**). Besides, we estimated the peaks would reach on April 16, April 5, April 21, and April 19 for Italy, Spain, Germany, and France, respectively, if the authorities took no measures, or would reach on March 11, June 7, March 15, and March 15, respectively (**Fig 4**), if lockdown was taken.

Finally, we performed a series of sensitivity analyses to test the robustness of our results by varying latent and infectious periods, transmission ratio between unascertained and ascertained cases, duration from illness onset to hospitalization, and hospitalized period (**Table 3**). Overall, the proportion of unascertained cases *r* maintained among different sensitivity analyses, except for S5, whose results (*i*.*e*.,0.40, 0.15, 0.31, 0.30 for Italy, Spain, Germany, France, respectively) were much lower than that of the main analyses. We noticed that the varying latent period affected the estimates of *b* slightly. A slight increase in the estimates of *b* and *R*_*0*_ was observed when the latent period increased from 4.6 days to 6 days. Nevertheless, doubling the infection period will moderately affect the estimates of *b* and *R*_*0*_. When assuming the transmission ratio was 0.5, only the estimates of *b* changed slightly (*i*.*e*., 0.48, 1.91, 0.77, 0.79 increase of *b* for Italy, Spain, Germany, France, respectively). The estimates of parameters were very close to the results of the main analyses when adjusting the *D*_*h*_ and *D*_*q*_ (*i*.*e*., sensitivity analyses S6-8).

## Discussion

Taking account of unascertained cases, hospitalized cases, and population movement, we herein modelled the initial stage of the epidemiological trend of COVID-19 based on the extended SEIR model for the four most serious countries in Europe, namely, Italy, Spain, Germany, and France. One of our major findings was that a majority part of infected cases was unascertained, exactly 41%, 78%, 54%, and 55% for Italy, Spain, Germany, and France, suggesting high covertness of COVID-19. The epidemic situation is severe in these countries, and much stricter and more efficient countermeasures, such as national lockdowns, are appealed to be implemented to slow down the spread of the epidemic as soon as possible.

When comparing the epidemiological features across these four countries, a relatively higher transmission rate of 2.83 (2.72–2.95) and a lower ascertainment rate of 0.22 (0.19–0.25) in Spain reflected a faster spread of the epidemic. It was in line with the fact that Spain has ranked the second most severely infected European country with more than 50,000 cumulative confirmed cases and a high mortality rate of 7.21% by the middle of March [12]. Comparing the transmission rate with that of the early epidemic stage in Wuhan, China (1.75 (1.71–1.80)) [11], Germany (1.91 (1.84–1.98)) and France (1.89 (1.82–1.96)) were slightly higher, while Italy (1.67 (1.64–1.71)) was a little lower and Spain was noticeably higher (2.83 (2.72–2.95)). It reflected a generally unideal awareness of prevention and control status across these countries, as it has been a long time since the signal of serious epidemic was sent. When transformed to the basic reproduction number *R*_*0*_, the differences across the countries were more obvious. Fixing the fraction of asymptomatic infectious that become reported symptomatic infectious at 0.6, Magal et al. utilized a similar modified SEIR model and reported basic reproduction number *R*_*0*_ to be 3.79, 4.21 and 4.45 for Italy, Germany, and France, respectively [27]. It seemed that our estimates of *R*_*0*_ are close to their results. The high transmission rate, low ascertainment rate, and high *R*_*0*_ for these countries indicated the severity of the COVID-19 epidemic. Efficient intervention measures, as well as self-administered protective actions, were essential to reduce the transmissibility of the epidemic and thus narrow the outbreak scope and prevent further burden.

We found the scale of the epidemic in these countries was all greatly reduced when strict countermeasures were imposed (*e*.*g*., quarantine, social distancing, and efficient case treatment) to retard the transmissibility from 100% to 25%. A smoother curve and a more delayed arrival of the peak of incidence came with a larger reduction of transmissibility, suggesting that we could win a window of opportunity for epidemic control and medical support if strong interventions were imposed. Notably, when the government reduced 75% transmissibility, the maximum number of new cases in four countries all strikingly dropped. Under this situation, Germany and France would confront a plateau in epidemic control, while the number of new cases of Spain would still be climbing for about 3 months, implicating that more forceful countermeasures are required in these countries to improve the situation. Yet Italy would welcome a downtrend, which could greatly reduce the medical burden.

Results of sensitivity analyses suggested that the estimations of *b*, *r*, *R*_*0*_ using the extended SEIR model were relatively robust to the inputs of most parameters, except for *α*, *γ* and *D*_*i*_. Regarding the *α*, there was no solid evidence to date that the transmissibility of the unascertained cases is stronger or weaker than that of the ascertained cases. Thus, it was reasonable to assume the same transmissibility for unascertained and ascertained cases. *γ*, which represented the ratio of unascertained cases to ascertained cases at the start point, was a sensitive parameter for the estimation of *r*. A recent study leveraged a capture-recapture method to estimate the hidden and total cases of COVID-19 for Italy, Germany, Spain, and other European countries [28]. The average ratio of the total estimated cases to the observed cases was around 2.3 (*i*.*e*., for every observed patient, there were 1.3 infected cases unascertained), stably. Our parameter setting of *γ* neared to the above results. That the estimates of the infectious period *D*_*i*_ in different researches varied in a wide range due to the diverse term definitions and model hypotheses [29] limited the accuracy of estimates for *b* and *R*_*0*_. As worldwide studies about COVID-19 deepen, we could expect to gradually eliminate uncertainties, for example, about differences of epidemiological parameters between different populations, therefore helping us to choose more accurate initial inputs for modelling.

We noticed that the observed data of Germany didn’t fit the model very well, which showed a relatively obvious fluctuation away from the supposed exponential distribution trend. We considered two potential reasons to account for this. In one respect, we assumed that the daily reports reflected the actual situation of infection, whereas, in fact, the accuracy of the data was limited by the detection capability and efficiency of the local health department in the initial stage of the epidemic. Additionally, there existed a situation where the cases confirmed the day before were reported the next day, which may interpret the unfitness to some extent.

There are still some limitations in this study. First, we did not consider the infectivity of cases in the latent period, although some current studies pointed out that pre-symptomatic transmission may occur [26,30–32]. Second, in the absence of the onset date of cases, we replaced it with the confirmed date, which may influence the parameter estimation. Third, the removed (*R*) compartment in the model contained both recovered and dead cases, and the few dead cases in the early stage did not fit the model as other studies [11,19]. Actually, we found the high covertness of COVID-19 and a large proportion of unascertained cases, which may suggest a lower death rate than that of the authority reported. Additionally, we assumed the recovered cases in the removed (*R*) compartment would not get infected again in the period we studied, since they have obtained the antibody [33–35]. Fourth, the assumption that all travelers into the country are susceptible may not hold, because the susceptible, latent, asymptomatic infected, or removed may also exist among the travelers. However, the proportion of the latent, asymptomatic infected, or removed relative to susceptible among travelers is small during the initial stage of COVID-19. This inaccurate assumption may not substantially affect our main results.
